# Addressing the Covid-19 Burden on Medical Education and Training: The Role of Telemedicine and Tele-Education During and Beyond the Pandemic

**DOI:** 10.3389/fpubh.2020.589669

**Published:** 2020-11-27

**Authors:** Divyansh Sharma, Sonu Bhaskar

**Affiliations:** ^1^Pandemic Health System REsilience PROGRAM (REPROGRAM) Global, Sydney, NSW, Australia; ^2^Neurovascular Imaging Laboratory, Clinical Sciences Stream, Ingham Institute for Applied Medical Research, Sydney, NSW, Australia; ^3^South Western Sydney Clinical School, University of New South Wales (UNSW), Sydney, NSW, Australia; ^4^Department of Neurology and Neurophysiology, Liverpool Hospital and South West Sydney Local Health District (SWSLHD), Sydney, NSW, Australia; ^5^NSW Brain Clot Bank, NSW Health Statewide Biobank and NSW Health Pathology, Sydney, NSW, Australia

**Keywords:** medical education, training, remote delivery, tele-education, telemedicine, technologies, digital humanities, Coronavirus Disease 2019 (COVID-19)

## Abstract

Medical students are the future of sustainable health systems that are severely under pressure during COVID-19. The disruption in medical education and training has adversely impacted traditional medical education and medical students and is likely to have long-term implications beyond COVID-19. In this article, we present a comprehensive analysis of the existing structural and systemic challenges applicable to medical students and teaching/training programs and the impact of COVID-19 on medical students and education. Use of technologies such as telemedicine or remote education platforms can minimize increased mental health risks to this population. An overview of challenges during and beyond the COVID-19 pandemic are also discussed, and targeted recommendations to address acute and systemic issues in medical education and training are presented. During the transition from conventional in-person or classroom teaching to tele-delivery of educational programs, medical students have to navigate various social, economic and cultural factors which interfere with their personal and academic lives. This is especially relevant for those from vulnerable, underprivileged or minority backgrounds. Students from vulnerable backgrounds are influenced by environmental factors such as unemployment of themselves and family members, lack of or inequity in provision and access to educational technologies and remote delivery-platforms, and increased levels of mental health stressors due to prolonged isolation and self-quarantine measures. Technologies for remote education and training delivery as well as sustenance and increased delivery of general well-being and mental health services to medical students, especially to those at high-risk, are pivotal to our response to COVID-19 and beyond.

## Introduction

The COVID-19 pandemic is a public health crisis with enormous and diverse social, economic and health consequences (1). Vulnerable and minority groups are being disproportionately impacted, often due to underlying social inequities, health disparities, and comorbidities (2, 3). Medical students are also vulnerable, particularly those from underprivileged or minority backgrounds. However, the plights of medical students in COVID-19 has drawn limited attention. Our previous reports highlighted that frontline medical professionals are vulnerable to mental illness or psychological distress (2, 4, 5). However, medical students are not considered part of the healthcare workforce, lacking access to COVID-19 mental health support available to healthcare workers. We postulate that medical students are especially vulnerable during the pandemic and hence require additional and tailored support, distinct from the general population or medical professionals. Whilst in specific instances or institutions, final year medical students' induction to the healthcare workforce has been expedited to meet increased demands during COVID-19, it's poignant that those in formative years of their education and clinical training have also been removed from structured in-person clinical environments (6, 7). The aim of this article is to critically evaluate the impact of COVID-19 on medical education, training and medical students; and to make targeted recommendations to maintain continuity and support mental health, well-being and education needs of affected students.

## Methods

Relevant literature was identified via PubMed and Medline review, including original, opinion and perspective articles, topic reviews, official national medical associations/bodies and societal guidelines and media sources. The PubMed/Medline search was performed using the keywords “Medical Students,” “COVID-19” and “Medical Education” until July 31, 2020. The PICO template, with the population (medical students), intervention (COVID-19), comparator (standard medical education pre-COVID-19) and outcome (impact on medical students/education and changes adopted due to COVID-19), was used. The literature was examined to critically analyse existing structural and systemic challenges of medical education, with an emphasis on the use of technologies such as telemedicine or remote education, and formulate a synthesis on the impact of COVID-19 on medical education, students and training. Appropriate articles relevant to COVID-19 were included in this synthesis. Medical students who are especially vulnerable, such as those with pre-existing mental illness, disadvantaged backgrounds, overseas medical students and those in under-resourced settings are considered and impact of COVID-19 on these subgroups are presented. We also provide targeted recommendations to address acute and systemic challenges during and beyond COVID-19.

## Results

### Impact of COVID-19 on Medical Students

Medical students are at increased risk of mental or psychological disorders, with a significantly higher prevalence of depression, depressive symptoms and suicidal ideations relative to the general population (8) (Table 1). Disruptions in traditional medical education and training due to COVID-19 have increased risk of poor mental health among medical students worldwide (Table 2) (8–16). The mental health burden could be exacerbated in those with pre-existing mental illness (4, 42). Concerns around inadequate skill development due to suspension of hospital placements, ambiguity around future prospects and subsequent financial implications have been reported. Poor health behaviors, sleep deprivation during COVID-19 and pre-existing chronic diseases among medical students could adversely affect physical and mental health (29), with cardiovascular disease, diabetes, obesity and chronic neurological comorbidities associated with increased risk of hospitalization and severe illness due to COVID-19. Targeted support and specific recommendations for these subgroups have been made elsewhere (2, 5).

**Table 1:** Data illustrating the burden of mental health in medical students.

**Parameter**	**General population (%)**			**Medical students (Pre-COVID-19) (%)**		
	**Australia (9)**	**UK (10)**	**USA (11)**	**Australia (12)**	**UK**	**USA**
Depression	4.1^*^ (Depressive episode)	3.3^***^	7.10^*^ (Major depression)	8.1^#^	9.7^###^^,^ ^***^ (HADS ≥ 8 (13)	58.2^*^ (14)
Anxiety disorders	14.4^*^ (include OCD and PTSD)	5.9^***^ (GAD)	19.1^*^	7.5^#^	38.4^###^^,^ ^***^ (HADS≥8) (13)	
Suicidal thoughts/ideations	2.3^*^	5.4^*^	4.3^*^	19.2^#^	14.9^##^ (15)	9.4^*^ (14)
Eating disorders			BED: 1.2^*^ BN: 0.3^*^ AN: 0.6^**^			
OCD	1.9^*^	1.3^***^	1.2^*^			
PTSD	6.4^*^		3.6^*^			
Substance use disorders	5.1^*^	Hazardous drinking (AUDIT>8): 19.4^*^ Drug dependence: 3.1^*^		**Alcohol use** Moderate: 19.0^#^ High: 4.0^#^ **Daily Use of:** Tobacco: 0.9^#^ Prescription Drugs: 2.6^#^ Illicit Drugs 1–6 times/week: 0.5^#^	Hazardous Drinking (AUDIT>8): 50.1^###^^,^ ^#^ (13) Smokers: 3.6^###^, (13) Other Substances^*^ (13) M1: 26.4 M2: 28.4 M3: 23.7	Alcohol abuse/dependence: 32.4 (16)

*BED, Binge Eating Disorder; BN, Bulimia Nervosa; AN, Anorexia Nervosa; PTSD, Post-Traumatic Stress Disorder; OCD, Obsessive Compulsive Disorder; GAD, Generalized Anxiety Disorder; HADS, Hospital Anxiety and Depression Scale; M1, 1st Year Medical Students; M2, 2nd Year Medical Students; M3, 3rd Year Medical Students*.

* *Past Year Prevalence*.

** *Lifetime Prevalence*.

*** *Past Week Prevalence*.

# *Current Prevalence*.

## *During Medical Studies*.

### *Calculated from study data*.

*Note the differing reporting methods and sample sizes. However, it is well-recognized that medical students are at greater risk than the general population. Recent evidence from the COVID-19 pandemic suggests that this is contributing to worsened mental health outcomes in this already vulnerable population; see Table 2 (25, 29, 41)*.

**Table 2:** Impact of COVID-19 on medical education, training and mental health of medical students and trainees.

**Study**	**Country/continent or region**	**Population**	**Variables/challenges**	**Impact/assessment**
Olum et al. (17)	Uganda/Africa	*n* = 741 1st to 5-year medical students	Knowledge, attitude and practices	Good knowledge, attitude and practices. 80% of students willing to participate in frontline care if required.
Nguyen et al. (18)	Vietnam/Asia	*n* = 5423 Medical students from eight different medical schools	Fear of COVID-19 (via the validated fear of COVID-19 scale)	Better health literacy, older age, later academic years, male gender and better financial status were protective from fear. Those with greater fear scores were more likely to smoke and drink at an unchanged or higher level than before the pandemic.
Flotte et al. (19)	USA/North America	*n* = 57 Final year Medical Students	Students were graduated early and participated in the workforce as limited license physicians	Were able to be deployed in “pods” of 3–4 and provide support to physicians. Received positive feedback.
Compton et al. (20)	Singapore/Asia	*N* = 179 All medical students at a graduate entry medical school in Singapore	Return to the clinical setting	Approximately one-third of students did not wish to return to the clinical setting, with the major concern being negatively influencing patient outcomes.
Chandra et al. (21)	USA/North America	*n* = 67 Senior Medical Students	Concerns around the inability to partake in emergency medicine clinical environments	Online teleconferencing was used to give students the ability to carry out follow-ups with discharged patients. Students reported positive feedback and benefits to their clinical reasoning from discussing with staff members. Additionally, they were pleased with feeling worthwhile in the pandemic.
Collado-Boira et al. (22)	Spain/Europe	*n* = 62 Final year medical (23) and nursing (24) students	Willingness to participate in the health workforce Fears about infection, familial transmission, lack of PPE, confidence in terms of knowledge and skills and coping	85.5% of recipients voluntarily joined, with the major reason being a desire to help in the COVID-19 situation. There were profound fears in all domains, particularly with respect to familial transmission, their practical knowledge and skills, and coping with the death of patients.
Khanna et al. (25)	India/Asia	*n* = 2,355 Ophthalmologists and ophthalmologists-in-training	Impact on training or professional work. Financial Implications. Symptoms of depression using PHQ-9 validated scale	52.8% felt their training or professional would be seriously affected by COVID-19, 37% reported difficulties meeting financial commitments and 32.6% had some degree of depression.
Zingaretti et al. (26)	Italy/Europe	*N* = 115 Plastic surgery residents in Italy	Impacts of COVID-19 on didactic teaching and professional development	Whilst residents reported increased didactic activities compared to pre-COVID, the majority reported them as insufficient. Additionally, most reported their preparedness for operations as either “Not at all” or “Not Much.”
Taghrir et al. (27)	Iran/Middle East	*n* = 240 5th to 7th year Iranian Medical Students	Knowledge, preventive behaviors and risk perceptions surrounding COVID-19.	Good knowledge, and high rates of preventative behaviors. Risk perception was moderate but tended to vary between different groups.
Guadix et al. (28)	USA/North America	*n* = 127 1st to 4th Medical Students	Impacts of the COVID-19 pandemic on medical student attendance on neurosurgery training camps	Postponement and cancellation were widespread (76%), and there were profound concerns surrounding conferences and networking opportunities, clinical experience and board examinations. Interestingly, 1st and 2nd-year students wanted virtual mentorship to address this, whereas 3^rd^ and 4^th^ preferred virtual surgical skills workshops.
Li et al. (29)	China/Asia	*N* = 1,442 Health professional students (764 medical, 211 nursing, and 467 medical technology)	Factors associated with psychological distress during the COVID-19 pandemic	26.63% of students had psychological distress that was clinically significant, whilst 11.10% had a probable acute stress reaction. Those with childhood adversity, stressful life event experiences in the past year and internet addiction were at greater risk, whereas good family functioning was protective.
Aker et al. (30)	Turkey/Middle East	*N* = 1,375 Medical Faculty Students	Views surrounding the COVID-19 pandemic	Over half of the students used social media as their source of information, but the majority did not trust this. About half of the students were concerned about receiving education in locations where COVID-19 patients were treated and would not want internships in such locations.
Bhagavathula et al. (31)	Global (308 respondents from Asia)	*n* = 453 Healthcare workers (137 doctors and 134 medical students)	Determination of the knowledge and perceptions related to COVID-19	Most participants obtained their information and knowledge surrounding COVID-19 from social media. Knowledge surrounding incubation period and transmission were poor. Doctors were found to be more well-informed than allied health workers.
Garcia et al. (24)	USA/North America	*N* = 315 (Medical students and foreign medical graduates working at US medical schools)	Determining the impact of the COVID-19 pandemic on medical students considering/already transitioning to neurosurgical careers	Approximately 2/3 of respondents reported postponement of clinical placements and suspended in-person teaching. Greater than 50% of respondents reported reduced academic productivity. One in five first-year medical students reported that they are less likely to pursue neurosurgery as a career option. Student-focused webinars and student-focused sessions at upcoming neurosurgical conferences were favored by students as ways to address these issues.
Liu et al. (32)	China/Asia	*n* = 217	Mental health status of medical students in Wuhan, China	35.5% of students were in a state of depression and 22.1% anxiety. The majority of affected students had mild to moderate symptoms
Khasawneh et al. (33)	Jordan/Middle East	*n* = 1,404 1st to 6th-year medical students	Knowledge, attitude, perceptions, and precautions surrounding COVID-19	Most students obtained their information from social media for information about COVID-19. There was adequate knowledge and appropriate precautionary strategies were carried out.
Meo et al. (34)	Saudi Arabia/Middle East	*n* = 625 1st to 5th-year Medical Students	Psychological well-being, stress and learning behaviors.	Feelings of emotional detachment and disheartenment were prominent. Additionally, students felt their work performance and time spent studying was reduced.
Abbasi et al. (35)	Pakistan/Asia	*n* = 382 Medical and Dentistry Students	Attitudes and perceptions surrounding e-learning	Most students had negative perceptions surrounding e-learning and preferred face-to-face learning. Many students used their mobile devices for e-learning purposes.
Sethi et al. (36)	Pakistan/Asia	*n* = 290 Healthcare professionals	Impacts of the shutdown on daily lives and health	For academics, work-life balance issues were identified as online teaching was an addition to extensive clinical work. Ensuring mental health impacts were reported. For some clinicians in training, academic delays and subsequent financial impacts were a concern. There were concerns about the lack of PPE.
Ikhlaq et al. (37)	Pakistan/Asia	*n* = 384 Medical, dental, nursing and allied health students	Awareness and attitudes	A resounding majority were aware of the etiology, mode of transmission and possible symptoms, but in-depth knowledge was lacking. Medical and nursing students had better knowledge. Most students showed positive attitudes, but a substantial proportion had fears around familial transmission. There were also concerns around the government's ability to address the COVID-19 pandemic.
Lin et al. (38)	China/Asia	*n* = 2,086 Medical students from a single medical school in Fujian, China	Impacts of mass and social media on psychobehavioural responses to the COVID-19 pandemic	Both mass and social media exposure assisted in increasing positive attitudes and reducing emotional consequences and behavioral prevention barriers
Choi et al. (39)	United Kingdom	*n* = 440 Final year medical students from 32 UK medical schools	Impacts on student learning and confidence for their 1st year of training	Significant impacts on student's preparedness due to impacts on OSCEs, written exams and student assistantships. The latter also had confidence implications.
Çalişkan et al. (40)	Turkey/Middle East	*n* = 860 Final year medical students	Knowledge and perceptions toward COVID-19	Moderate knowledge. Those with better knowledge had lesser fear. Most students reported not having been trained until the pandemic hit Turkey. Many students also felt unprepared if required to assist in the emergency department.

### Impact of COVID-19 on Medical Education

Clinical clerkship is fundamental to medical students learning from more experienced practitioners, and becoming more independent and confident clinically (43). The Association of American Medical Colleges (AAMC) released guidelines recommending withdrawal of medical students from clinical environments after the COVID-19 outbreak (44). This was followed by suspension of clinical placements by several Australian medical schools to reduce risks associated with more personnel in clinical environments (45). This trend is true worldwide and has caused widespread concerns from medical students about their learning (46). Lost in-hospital clinical clerkships have caused fears surrounding deficiency in practical skills, training and “imposter syndrome” (17, 20, 46, 47). This extends beyond medical students, with surgical trainees reporting fears around preparedness for surgeries due to the suspension of elective surgeries and face-to-face training (26, 48).

Owing to the climate of uncertainty and limited clinical exposure, concerns surrounding progression through the medical course, training pathways and job prospects have been reported (45, 47, 49), including exploration of alternative career paths (24, 28). This also applies to those at advanced stages in their training, such as trainees and specialists-in-training (26, 28).

### Impact of COVID-19 on Medical Students From Vulnerable Groups

Impact on various segments of vulnerable populations within the broad medical students' community is presented below.

#### Students With Pre-existing Mental Health Issues

The pandemic has caused increased stressors, with social isolation measures particularly associated with, and resulting in, increased depression, anxiety and suicidal ideations, as well as poor health behaviors among medical students during COVID-19 (32, 34), and also medical specialists and trainees (23, 50). This issue is exacerbated by lower uptake of mental health services due to stigma surrounding mental health issues, confidentiality concerns, and the belief that medical students should be self-sufficient in addressing and coping with their mental health issues (51). Social isolation is known to be linked to psychological disorders and/or mental health issues in otherwise healthy individuals, and those with existing mental illness are especially vulnerable, with an increased likelihood of concomitant social factors that can worsen their vulnerabilities (52). These may include conversion disorders, acute stress disorders, mood disorders and frustration (25). Such issues can persist beyond periods of social isolation and are associated with longer-term disorders such as post-traumatic stress disorder symptoms and exacerbation of obsessive-compulsive disorder like symptoms (4), as reported after the severe acute respiratory syndrome outbreak (52). Additionally, medical students currently on psychotherapy or psychiatric interventions may face difficulty in the prescription refill and attending regular therapy sessions (52).

Recent studies show that whilst poor eating behaviors are being seen in the general population, this is more pronounced in those with pre-existing eating disorders (41, 53). Medical students may also be at increased risk of developing eating disorders due to academic and emotional stressors (54). Those with existing eating disorders may be vulnerable to COVID-19 infection and resultant anxiety concerning their health (55). Fears about inability to obtain foods consistent with individual meal plans presumably increases anxiety, and fears of relapse (41, 53). Increased social isolation, gym closures and social media use may also precipitate relapse, and body image concerns, causing withdrawal from engaging with others through video calls, leading to heightened isolation (55). Whilst video-based cognitive behavior therapy (CBT) has shown efficacy, the success of this in pandemic situations is largely unknown (56), with patients in the early transition feeling their quality of care was reduced. Individuals with childhood traumas are also more vulnerable to mental health impacts and warrant special consideration (29).

#### Students From Financially Disadvantaged Backgrounds

Telecommunications technology has provided an effective way to address gaps in learning caused by the pandemic. However, for those engaging in online learning, there may be inequities, and subsequent frustration and stress, as even in developed countries, not all students have access to the digital devices or infrastructure required to effectively partake in online learning (57). Moreover, those in remote and rural areas often have poor internet connections (58). Prolongation of the course length can have significant financial consequences and hence impact academic progression (18).

The widespread redundancies, job losses and closure of non-essential services due to COVID-19 have forced several students to resume work to support their families (57). This can negatively affect their ability to engage in online learning activities due to concurrent work responsibilities (18). Medical students from disadvantaged backgrounds reliant on public transport to commute also face greater infection risks.

#### Students in Developing Countries

Students in developing countries face unique challenges such as limited availability of online teaching resources for medical education (59). The frontline healthcare workers handling COVID-19 associated hospitalizations are limited in their ability to develop teaching resources (36). Given that online teaching modalities have been largely unexplored in many of these countries, these issues have been exacerbated (60). Poor internet access and stability are also reported, causing an unwillingness on students' part to transition to online teaching modalities, and negative perceptions surrounding use of telecommunications technology for learning (35). Perceived poor responses by governments and public leadership have also inflicted psychological stress among students (37).

#### Students From Minority Groups

Medical students belonging to racial, ethnic and linguistic minority groups face unique challenges due to systemic barriers (61, 62). A 2019 American study showed that white students were more likely to have grading disparities favoring them than minority groups (62). Implicit bias, along with factors such as inappropriate learning environments for minority groups, were reported to drive these disparities. Incorporation of concrete rubrics and marking criteria to limit subjectivity and implicit bias training programs to educate examiners may reduce these (62).

A 2020 study similarly found that underrepresented minorities, Asian and multiracial students, were more likely to be deprived of opportunities based on race than white students (7.3, 4.4, 3.6 vs. 1.5%, respectively) and be subjected to racially offensive comments (18.9, 12.9, 9.6 vs. 2.5%, respectively). Additionally, female students were more likely to be discriminated than males (28.2 vs. 9.4%) (63), and lesbian, gay and bisexual students were more likely to be mistreated than heterosexual students (43.5 vs. 23.6%) (63).

There are concerns that people from racial and ethnic minority backgrounds, already reported to have higher rates of infection and racial discrimination during the pandemic, will present later and at more advanced stages of disease due to fears of structural racism, thereby increasing clustering and community transmission risk (64–66). Systemic issues around underreporting of racial harassment also exist, contributing to increased mental health risks among minority groups, with beliefs existing that complaints pertaining to discrimination will not be taken seriously and/or addressed appropriately (67). To our knowledge, there is limited research into impacts of COVID-19 on the lesbian, gay, bisexual, transgender, queer and intersex (LGBTQI+) community. However, systemic inequalities and inequities are known to cause long-term stress, increasing vulnerability to negative mental health implications. Of particular concern is isolation and separation from trusted family and friends due to quarantine measures (68). Medical students are particularly reluctant to discuss sexual orientation, thereby raising concerns over their ability to seek help (61). Confidential telehealth services have sprung up worldwide and may assist students from minority groups.

#### International Medical Students

International medical students are particularly impacted, with several institutions shifting to online delivery methods soon after the start/resumption of the academic year (58). Resultantly, there is an increased risk of isolation and subsequent mental health issues, with an Australian study showing that international students have higher baseline depression risk than local students considering loneliness, anxiety and stress scores (69). Additionally, loss of employment, financial insecurity and lack of family support are significant, especially for international students not returning to their home countries (52), as social and family support may be protective against mental health sequelae (29). With university campuses closing down, accommodation may become an issue, exacerbated by job losses from closure of non-essential services (70).

For students having returned to their home countries, concerns surrounding academic progression are likely stressors, amidst new immigration measures including indefinite sealing of borders to non-citizens, temporary-residents or immediate family thereof by several countries (71). Variation in time zones during online learning for overseas courses or seminars may impact sleep cycles, with insufficient sleep associated with various mental disorders including depression (72). Once border restrictions are eased, and foreign students return to their host countries, it may not be feasible for them to return home to loved ones (73, 74). Travel restrictions could prolong course length and incur subsequent financial burden (70).

The inability to access clinical environments is particularly relevant, as engaging with patients and peers of their host country is pivotal to developing cultural competence and understanding socio-cultural norms, expectations and communication methods, along with language skills. Lack of this can be a stressor and contribute to imposter syndromes (75). Telecommunications technology may revive some degree of communication. However, non-verbal body language, pivotal in communication, cannot be adequately simulated (76).

### Medical Student Parents

Medical students who are parents may have hindered engagement in interactive learning via telecommunications due to a need to take care of children, which other students might not appreciate, causing feelings of isolation and exclusion, and frustration (77, 78). Additionally, individuals may feel caught between notions of service and personal responsibility to their family, which can have personal mental health implications pertaining to guilt (79).

## Discussion and Recommendations

The COVID-19 pandemic has not only presented acute challenges to medical education and students but also exposed systemic issues that merit consideration. Given the COVID-19 pandemic has had an exacerbated impact on vulnerable populations, including but not limited to students from vulnerable backgrounds; evidently, these students would need targeted interventions, while recognizing the unique challenges in the COVID-19 era and accounting for socioeconomic aspects. Technologies such as telemedicine and tele-education are emerging as important platforms in mitigating the devastating impact of COVID-19 (80–82). Given the acute impact vis-a-vis rising mortality globally (5, 83), and long-term effects of this outbreak that may last beyond the pandemic (2), strategies toward capacity building are necessary.

We provide recommendations to address the negative impacts of the COVID-19 pandemic on medical student mental health and well-being and to build sustainable healthcare and education systems beyond the pandemic.

### Technology in Remote Delivery of Medical Education and Training

#### Restructuring Teaching and Examination Processes

##### During COVID-19

COVID-19 has led to rapid uptake and development of online teaching to minimize disruption to student learning. Telecommunication technologies are an important component in this, with several institutions having implemented online teaching webinars, simulations and educational clinical skill videos (21, 46). Multimodal teaching approaches catering to various aspects of learning have been implemented (84), along with flipped learning methodologies, which involve students engaging with content prior to class and using later face-to-face time to clarify concepts. This is useful for teaching anatomy using online 3D modeling applications considering suspension of traditional cadaver-based anatomy demonstration at several institutions (85). Online teaching can be made more engaging and effective for students through interactive tools such as voting polls, chat functions and videos (86). Additionally, intensive anatomy and clinical skills workshops, building on online learning resources, can be run when students return to in-person teaching to address deskilling and imposter syndrome concerns (58). Virtual tools such as virtual reality simulations, homemade simulations and smartphone modalities could benefit surgical trainees (46, 87).

These approaches, reliant on effective use of telecommunications technology facilitate enhanced student pedagogy, and thus address the stressors of deskilling, progression and hindered knowledge. Involvement of students in telehealth to provide clinical exposure and help triage patients during the pandemic has been well-received and facilitates controlled patient exposure with feedback (21). Tele-health-based services to partially replace overseas elective placements, although not equivalent, may allow students to gain an enhanced understanding of another healthcare system (88).

Accelerated progression may help ease burdens on the healthcare system. However, immediate transition into clinical practice could instigate higher rates of work-related stress, and this needs to be monitored (89). Concerns around litigation also exist (90), and mandating indemnity guarantees before students are offered jobs could be a solution. Additionally, students can be recruited into hospitals they are familiar with, to facilitate easier and less stressful transitions (91). Increased repurposing of specialists into different roles to assist with the response to the pandemic, and the increasing reliance on telemedicine, sustained supervision and detailed training are necessary to facilitate a seamless transition (4).

Considering higher levels of baseline stress, anxiety, and mental health implications during the pandemic, and the recency of the changes to learning modalities having been implemented, variations in exam structure could be considered. Open book examinations provide an alternative that could reduce students' stress and anxiety, with some institutions also considering pass/fail grading (92, 93). Additionally, students need education about evidence-based medicine, research methodologies and reliable sources, as several students rely on social media for their information on the pandemic (30, 31, 33). Consequently, medical students can be involved in curating evidence-based recommendations and research, to deepen understanding of bias and confounding, and assist in fighting misinformation in the community (94, 95).

Finally, for those entering clinical environments, targeted training in addressing the unique needs of the COVID-19 pandemic would be necessary, with a recent systematic review suggesting a multimodal training approach, which improves student skills, knowledge and attitudes (96). This would also entail teaching around effective communication using telemedicine, including professionalism and catering for patients with different technological capabilities (97).

##### Beyond COVID-19

Flipped learning could be of great utility toward encouraging independent learning, an integral part of ongoing medical professional development. Due to limited access to cadavers for medical education, such learning methods may be necessary beyond the pandemic (98) and may provide students greater flexibility. Training or volunteering opportunities to work in infectious disease outbreak settings, particularly via teaching around effective telemedicine consultations, could be embedded into medical school curricula to develop student confidence and resilience should an epidemic occur in future (96, 97).

Considering minority groups, training examiners on the role of implicit biases may facilitate longer-term benefits in education and assessment of medical students, and in establishing equitable and fair training, which will indelibly influence students' mental health positively (62). Finally, the misinformation propagated through social media with regards to this pandemic illustrates the role for doctors to act as educators, which could be a key part of medical school curricula going forward (99). Medical students should also be encouraged to be proactive to gain knowledge about COVID-19, to increase awareness on and contest misinformation (100).

#### Skill Building

##### During COVID-19

Incorporating inclusive language in everyday practice is pivotal in preventing marginalization of patients from the LGBTQI+ community, who already experience increased levels of stress and fear (68). Mental health first-aid training can help medical students develop strategies to cope with stressors, and help reduce stigmatizing attitudes toward mental health in the broader community (101, 102), with the Australian Government announcing funding to this end (103).

Webinars by experts from various medical disciplines have set a benchmark toward upskilling students and trainees and maintaining their interest and motivation. Using staggered timings to overcome issues related to time zones has been highly effective and can contribute to a global sense of community (46, 104). One such initiative is a collaborative series of recorded seminars and accompanying associated modules by the American College of Surgeons Division of Education and Association for Surgical Education (105), which specifically assist medical students with core surgical knowledge (105). Numerous institutions have also successfully implemented volunteering initiatives including research, assisting hospital triage, contact tracing, and support hotlines to support medical services during the pandemic but also boost student morale as they develop skills and feel “useful” (21, 106, 107). Telehealth service forms the backbone of such initiatives, allowing students to develop skills safely (108, 109).

##### Beyond COVID-19

Evaluation of skills development programs and their effectiveness is critical. Should they prove beneficial, their incorporation into regular teaching through telecommunications technology could potentially positively influence medical student and trainee mental health in an accessible and convenient manner. Additionally, considering the unpredictability surrounding further spread of the virus, and future outbreaks, such programs may inform future methods of addressing pandemics (108).

### Telemedicine for Care of General Well-Being and Mental Health of Medical Students

#### Creation of Mental Health Support Networks

Supporting medical students and trainees should not be limited to times of crisis such as this pandemic, but this pandemic has brought this important issue to the forefront. We discuss various strategies that can foster mental health support networks for students harnessing technology as an enabler (95).

##### Mentoring groups

*During COVID-19*. The creation of mentoring groups for students can facilitate sharing of ideas, advice and combating feelings of isolation. This can be particularly beneficial for international students who have not returned home and may feel isolated, as well as minority groups (69). Such groups can be stratified, with senior students providing advice and guidance to junior students, which may help address stressors related to progression and encourage participation in other areas such as research and peer teaching (110). Discussions with other students can provide a sense of unity and reassure students that they are not alone in feeling “imposter syndrome,” with sharing of online resources such as 3D anatomical models also being beneficial (111). Students from various backgrounds, including international, minority and medical student parents can be placed together in groups to facilitate increased understanding of the various challenges others from different backgrounds face, which may assist with developing empathetic competence (111). Such mentoring doesn't need to be faculty-driven but can be led by medical students themselves, who can use existing social media networks and telecommunications to overcome barriers imposed by social distancing policies (104, 111–113).

*Beyond COVID-19*. Mentoring groups or networks developed during COVID-19 should be continued beyond this pandemic. This would provide students an opportunity to interact with others, and obtain advice and guidance about study, research, and future training prospects (24, 110). Also, the social media-enabled hashtag support networks should be encouraged and propagated, with the “pay it forward” attitude (104, 112).

##### Tele-Psychiatry and Support Services

*During COVID-19*. Considering increasing reports of mental health consequences of the pandemic on medical students and trainees, telemedicine can be harnessed to provide constant support to these individuals and resultantly safeguard our future generations of medical professionals from longer-term sequelae. Confidentiality would be crucial to such a system, as many individuals, particularly those from minority groups, are reluctant to share their vulnerabilities (61). Additionally, training psychologists and psychiatrists involved in this service about unique demands of different groups will be critical to providing personalized care (74). Several telepsychiatry and support services have been started worldwide (Table 3).

**Table 3:** Various support services available to doctors and medical students in various countries and regions^*^.

**Country**	**Name**	**Website**	**Hotline number**	**Mode of delivery**
Australia	Drs4Drs support service (114)	http://www.drs4drs.com.au/	1300 374 377 (1300 DR4 DRS)	Phone
United Kingdom	NHS staff support line (115)	https://people.nhs.uk/help/	0300 131 7000	Phone
			Text FRONTLINE to 85258	Text Message (24 h support)
	NHS virtual staff common room (115)		N/A	Zoom Video Conferencing Platform (groups of 10, hosted by practitioners)
	Project5 well-being support service (116)	https://www.project5.org/getsupport	N/A	Online-linked with a “supporter” who is either a Coaching or Mental Health Professional
Canada	Wellness support line (117)	https://www.cma.ca/supportline	Newfoundland and Labrador, Nunavut, Saskatchewan and Yukon: 1-844-675-9222	24/7 hotlines with dedicated Physician and Family Support Program Physicians, who can link callers with relevant services for them
			Ontario: 1-800-851-6606	
			Nova Scotia: 1-855-275-8215	
	Physician health program of British Columbia 24 h helpline (118, 119)		British Columbia and Prince Edward Island: 1-800-663-6729	24/7 hotlines with dedicated intake counselors who can connect callers with physician support
	Quebec physicians' health program (PAMQ) Telephone (117, 119)		Montreal: 1-514-397-0888 Rest of Quebec: 1-800-387-4166	Hotline which allows connection to Physician Advisors
	Alberta Medical Association–Physician and family support program (117, 120)		Alberta: 1-877-SOS-4MDS (767-4637)	24/7 hotline with dedicated Physician and Family Support Program Physicians, who can link callers with relevant services for them
	Doctors Manitoba–Physician and family support program (117)		Manitoba: 1-844-4DOCSMB (436-2762)	Confidential 24/7 hotline
India	Indian Medical Association Psychosocial Counseling Helpline (121)	https://www.ima-india.org/ima/	+91 9999 11 6375 +91 9999 11 6376	Helpline operational 9 am to 9 pm daily
United States of America	Physician Support Line (122)	https://www.physiciansupportline.com/	1 (888) 409-0141	Confidential helpline for physicians run by volunteer psychiatrists. Open 7 days a week: 8:00 AM−1:00 AM ET.

* *This is not exhaustive, and the information provided was current at the time of writing*.

Additionally, CBT has been found to have utility for both depression and generalized anxiety disorder in university students. Although online CBT is not as effective as face-to-face equivalents, COVID-19 precautions and restrictions mean it is a useful way provide students with positive coping skills, and can bypass fears of stigma within medical student populations. Engagement has been problematic with online platforms in the past, and thus constant appraisal and remodeling are critical (123–125).

*Beyond COVID-19*. Maintenance of such telemedicine services provide medical students and trainees with a convenient and accessible outlet for any mental health concerns they may have and resultantly can assist in early identification or prevention of longer-term impacts of known stressors associated with the medical profession (95). Additionally, normalizing health-seeking behaviors through supportive rhetoric can help overcome barriers related to stigma (51).

##### Increase Accessibility to Support Services

*During COVID-19*. Support services are being implemented for vulnerable students. For instance, the NSW state government in Australia has provided funding for crisis accommodation for international students under financial duress due to the COVID-19 pandemic (126). Informing students to whom such support mechanisms are relevant is pivotal, with use of social media, targeted mail-outs, and newsletter segments being possibilities.

*Beyond COVID-19*. Appraising and optimizing such targeted communication with student subgroups can make them feel more valued and allow early access to any support required. Additionally, incorporating student input into developing programs can make these more effective and targeted to their needs.

Minority groups may be unlikely to make complaints about harassment and bullying for fear of their complaint not being taken seriously or retaliation. To address this, and increase the accessibility of support, public statements of zero tolerance for bullying, harassment and discrimination should be made. Additionally, diversification of staff and complaints committees and incorporation of lived experience members may be useful in highlighting to students that their viewpoints and challenges will be respected and that they will get fair redressal for untoward experiences or incidents (67, 127).

In conclusion, medical students, the future of our healthcare system, are vulnerable during the current pandemic, with subgroups of medical students from specific backgrounds more impacted. Targeted support for these subgroups, and students overall, is warranted. COVID-19 has exposed systemic issues within our healthcare and education systems. Recognizing these issues and developing strategies to combat them is pivotal to our response to an infection outbreak in the future.

## Data Availability Statement

The original contributions presented in the study are included in the article/supplementary materials, further inquiries can be directed to the corresponding author.

## Author Contributions

SB contributed to the planning, draft and revision of the manuscript supervision of the student, and encouraged DS to investigate and supervised the findings of this work. SB and DS wrote the first draft of this paper. Both authors contributed to the revision of the manuscript and approved the final draft of the manuscript.

## Conflict of Interest

The authors declare that the research was conducted in the absence of any commercial or financial relationships that could be construed as a potential conflict of interest.
